# Emerging roles of exosomal miRNAs in diabetes mellitus

**DOI:** 10.1002/ctm2.468

**Published:** 2021-06-27

**Authors:** Xiaoyun He, Gaoyan Kuang, Yongrong Wu, Chunlin Ou

**Affiliations:** ^1^ Department of Pathology, Xiangya Hospital Central South University Changsha Hunan 410008 China; ^2^ Departments of Ultrasound Imaging, Xiangya Hospital Central South University Changsha Hunan 410008 China; ^3^ Department of Orthopedics The First Affiliated Hospital of Hunan University of Chinese Medicine Changsha Hunan 410007 China; ^4^ Postdoctoral Research Workstation Hinye Pharmaceutical Co. Ltd Changsha Hunan 410331 China; ^5^ Hunan university of Chinese Medicine Changsha Hunan 410208 China

**Keywords:** diabetes mellitus, exosomes, insulin resistance, miRNAs, therapy

## Abstract

Exosomes are small extracellular vesicles 40–160 nm in diameter that are secreted by almost all cell types. Exosomes can carry diverse cargo including RNA, DNA, lipids, proteins, and metabolites. Exosomes transfer substances and information between cells by circulating in body fluids and are thus involved in diverse physiological and pathological processes in the human body. Recent studies have closely associated exosomal microRNAs (miRNAs) with various human diseases, including diabetes mellitus (DM), which is a complex multifactorial metabolic disorder disease. Exosomal miRNAs are emerging as pivotal regulators in the progression of DM, mainly in terms of pancreatic β‐cell injury and insulin resistance. Exosomal miRNAs are closely associated with DM‐associated complications, such as diabetic retinopathy (DR), diabetic nephropathy (DN), and diabetic cardiomyopathy (DCM), etc. Further investigations of the mechanisms of action of exosomal miRNAs and their role in DM will be valuable for the thorough understanding of the physiopathological process of DM. Here, we have summarized recent findings regarding exosomal miRNAs associated with DM to provide a new strategy for identifying potential diagnostic biomarkers and drug targets for the early diagnosis and treatment, respectively, of DM.

## Graphical abstract

Recent studies have closely associated exosomal microRNAs (miRNAs) with various human diseases, including diabetes mellitus (DM), which is a complex multifactorial metabolic disorder disease. In the diabetic condition, exosomal miRNAs are taken up by recipient cells, where they exert their biological function and thereby modulate the progression of DM‐associated complications, including diabetic retinopathy (DR), diabetic macrovascular complications (DMCs), diabetic nephropathy (DN), diabetic foot ulcer (DFU), diabetic peripheral neuropathy (DPN), and diabetic cardiomyopathy (DCM).

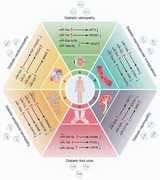

**Abbreviations**
EVs: extracellular vesicles
HUVECs: human umbilical vein endothelial cells
lncRNAs: long noncoding RNAs
miRNAs: microRNAs
MSCs: mesenchymal stem cells
TGF: transforming growth factor

## 1 INTRODUCTION

Diabetes mellitus (DM) refers to a class of metabolic disorders characterized by hyperglycemia. DM results from defective insulin secretion or insulin resistance (IR) caused by genetic or environmental factors.^1^, ^2^, ^3^ With the rapid increase in population growth, aging, and urbanization, and the increased prevalence of obesity and physical inactivity in recent years, DM has become a global health problem. Epidemiological studies have indicated that the global prevalence of DM is expected to increase from 4% in 1995 to 5.4% in 2025.^4^ The annual health care costs for the predicted 693 million people with DM by 2045 are estimated to be approximately US$850 billion.^5^ In recent years, DM has become the third most common noninfective disease, following cardiovascular diseases and tumor‐related diseases.^6^ Although significant progress has been made in the understanding and therapy of DM, the related morbidity and mortality rates have continued to increase. Effective biomarkers for the early diagnosis, progression monitoring, and targeted therapy of DM are urgently needed.^7^

Various extracellular vesicles (EVs) that are stably present in human fluids have recently been described.^8^, ^9^ EVs can be classified, based on their size, as small EVs (sEVs, diameter <200 nm) and large EVs (lEVs, diameter >200 nm). Exosomes are a class of sEVs that have a diameter of approximately 40–160 nm and are derived from almost all types of human cells.^10^, ^11^, ^12^, ^13^ Exosomes are widely present in all types of human body fluids, such as saliva, ascites, breast milk, cerebrospinal fluid, urine, and semen.^14^ They can carry RNA, DNA, lipids, proteins, and metabolites^15^ and are important for the transmission of information between cells.^16^ In the era of precision medicine, increasing attention is being paid to the accurate diagnosis and treatment of diseases.^17^ The use of exosomes for the effective diagnosis and treatment of diseases is emerging as a research focus. The potential of exosomes in precision medicine is based on their ubiquity in the body and their ease of acquisition.^18^, ^19^

With the completion of the Human Genome Project and the beginning of the postgenomic era, noncoding RNAs (ncRNAs) have aroused great interest in various research areas. MicroRNAs (miRNAs) are a type of ncRNA that are approximately 22‐nucleotide (nt) long and are encoded by endogenous genes.^20^ miRNAs are widely found in plants, animals, and some viruses. They function in transcriptional or posttranscriptional regulation by binding to the untranslated regions (UTRs) of target mRNAs, which participate in regulating the physiological and pathological processes of the human body.^21^ miRNAs are more stable and specific in tissues and blood than long noncoding RNAs (lncRNAs) and mRNAs because of their shorter sequences.^22^ Interestingly, miRNAs can be encapsulated in exosomes, which carry them to and release them into target cells or tissues, where their biological regulatory effects occur. Most cells in the human body can secrete exosomes containing miRNAs. For example, approximately 100 miRNAs have been detected in the exosomes secreted by mast cells.^23^, ^24^ Exosomal miRNAs not only participate in normal physiological processes but are also involved in the occurrence and development of various diseases,^25^, ^26^, ^27^ including DM. Exosomal miRNAs are emerging as pivotal regulators in the development and progression of DM. Moreover, because of their specificity and sensitivity, exosomal miRNAs released into the humoral circulation have potential for use as DM markers.^28^, ^29^ Further investigations of the mechanisms of action of exosomal miRNAs and DM will be valuable for thoroughly understanding the physiopathological process of DM. This review focuses on the latest findings regarding the roles and regulatory mechanisms of action of exosomal miRNAs in the development of DM. This information provides a theoretical basis for the potential use of exosomal miRNAs as DM therapeutic targets.

**HIGHIGHTS**

- Exosomal miRNAs are essential regulators of almost every aspect of human diseases.
- Exosomal miRNAs are emerging as pivotal regulators of DM progression, mainly in terms of pancreatic β‐cell injury and insulin resistance.
- Exosomal miRNAs are closely associated with DM‐associated complications, such as diabetic nephropathy, diabetic retinopathy, and diabetic cardiomyopathy.
- Exosomal miRNAs are promising biomarkers and targets for the diagnosis and therapy of DM.

## 2 BIOLOGICAL CHARACTERISTICS OF EXOSOMAL MIRNAS

Exosomes were first discovered by Trams et al. in 1981. The authors observed small membranous vesicles in the supernatants of tumor cells cultured in vitro and termed them exosomes.^30^ At that time, it was thought that exosomes function only as a waste disposal system for cells. With the increasing developments in electron microscopy technology, Johnstone et al. isolated exosomes for the first time during the study of reticulocyte maturation and found a transfer receptor on the exosome membrane.^31^ Further research has demonstrated the involvement of exosomes in diverse biological processes, such as antigen presentation, immune response, tumor growth, and cell differentiation.^32^

Exosomes are small vesicles secreted by various cells. They are composed of a lipid bilayer and have an approximate diameter of 40–160 nm. Exosomes are multivesicular bodies (MVBs) formed from endodermal buds.^33^, ^34^ Exosome formation involves encounter with the cells membrane, enzyme modification, fusion with the cell membrane, and subsequent release from the cell. Almost all mammalian cells can produce and release exosomes, including blood cells (such as T lymphocytes, B lymphocytes, mast cells, and platelets), dendritic cells, and other cells (including epithelial cells, neurons, and astrocytes).^35^, ^36^, ^37^, ^38^, ^39^ Exosomes are widely present in various body fluids, including urine, milk, saliva, and blood. They harbor proteins, lipids, nucleic acids, and other substances. The circulation of exosomes in body fluids allows them to enter nearby or distant target cells and plays a role in the delivery of their payloads via direct fusion, endocytosis, and binding of receptor ligands.^40^, ^41^, ^42^ Exosomes are a conduit of biological signal transmission between cells. They affect the physiological state of cells and are closely related to the occurrence and progression of various diseases.^43^ The prevalence and stability of exosomes in various biological processes as well as the development and refinement of analytical tools and techniques to quantify and identify the characteristics of their constituents have spurred the recognition of exosomes as potential biomarker candidates for disease diagnosis and prognosis.

After entering a target cell, an exosomal miRNA can directly bind to the 3′ UTR of the target mRNA, inhibiting the expression of the target gene and its downstream molecules. Exosomal miRNAs can also be combined with “sponges,” such as lncRNAs, to play a physiological regulatory role. Exosomal miRNAs can be transferred to target cells or target organs to regulate gene expression and can also be stably stored in circulating fluid, protected by their vesicle structure; thus, they can act as biomarkers to reflect disease progress.^44^, ^45^ Accumulating evidence indicating that exosomal miRNAs are important in the development of diseases has indicated their potential value as biomarkers for disease diagnosis, prognosis, and personalized treatment.

## 3 DYSREGULATION OF EXOSOMAL MIRNAS IN DM

The prevalence of DM has continued to increase over the past 50 years, with the disease having spread from Western countries to Africa, Western Pacific, and Asia.^5^ In recent years, DM has quickly become a global health problem. According to clinical characteristics, DM can be divided into four types: type 1 DM (T1DM), type 2 DM (T2DM), gestational DM (GDM), and other specific types. Chronic hyperglycemia in DM is usually associated with long‐term damage, dysfunction, and failure of multiple organ systems, especially the eyes, heart, kidneys, nerves, and blood vessels.^46^, ^47^ The consequence can be a series of DM‐associated complications, including diabetic retinopathy (DR), diabetic macrovascular complications (DMCs), diabetic nephropathy (DN), and diabetic cardiomyopathy (DCM), and diabetic foot ulcer (DFU). DM‐related morbidity and mortality can be reduced by the regular screening, early detection, and appropriate treatment of chronic complications that are known to lead to it.^48^, ^49^ The identification of appropriate and effective biomarkers to prevent and treat DM and its associated complications is an urgent goal.

**FIGURE 1:**
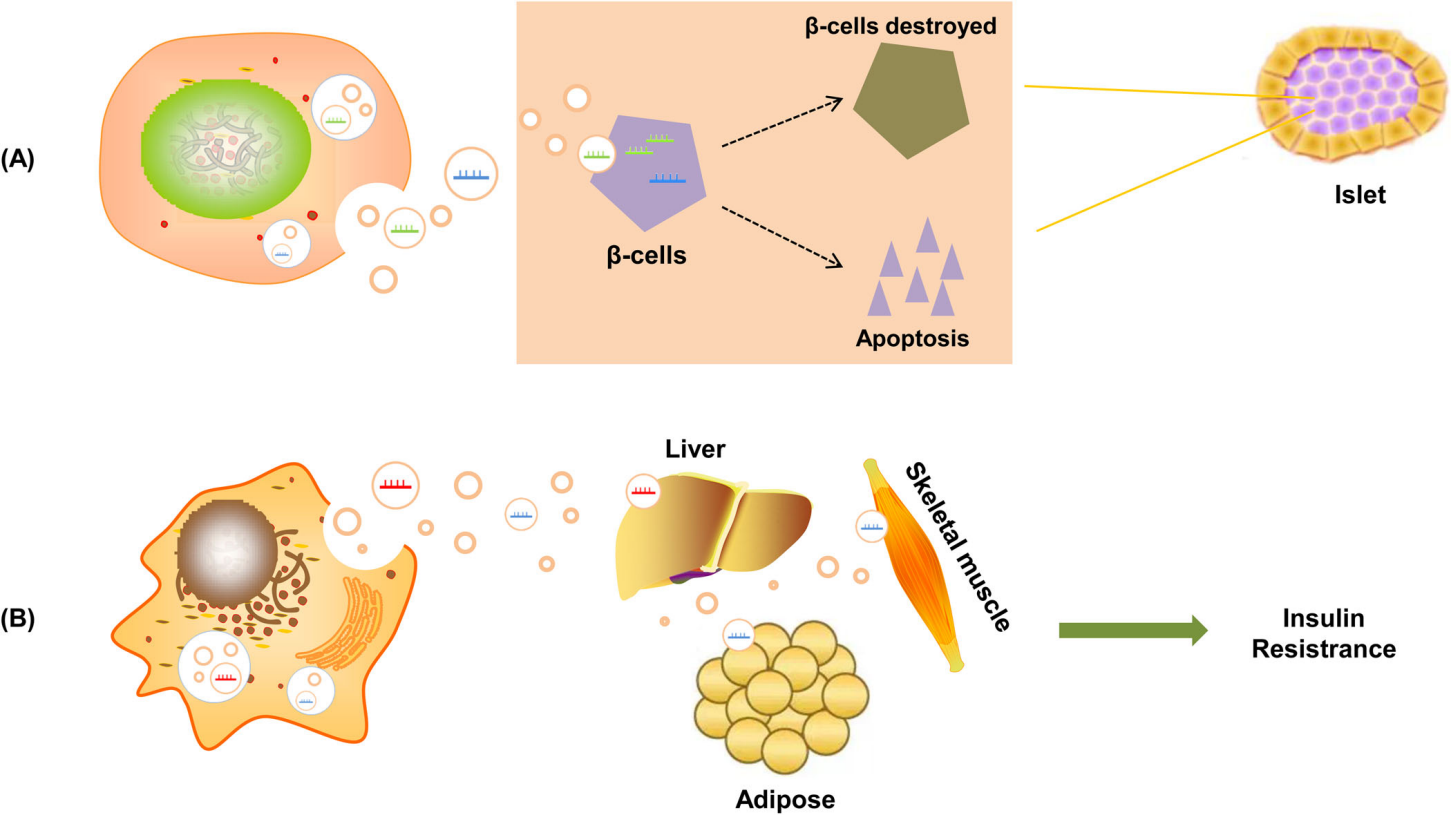
Exosomal miRNAs regulate the pathological process of DM. (A) Secretion of cell‐derived exosomal miRNAs promotes the destruction of pancreatic islet β‐cells and β‐cell apoptosis. (B) Secreted exosomal miRNAs induce insulin resistance in the main insulin‐sensitive organs (skeletal muscle, liver, adipose)

With the development of next‐generation sequencing technologies, increasing numbers of miRNAs are being identified. miRNAs have thus been found to be important regulatory molecules in the development of DM as well. miRNAs such as let‐7, miR‐223, miR‐29, and miR‐103 can regulate metabolic disorders, such as DM, through multiple pathways, such as the regulation of glycolipid metabolism, liver glycogen metabolism, and insulin secretion.^50^, ^51^, ^52^ Exosomes also play an important role in DM development and progression.^53^ miRNAs harbored in exosomes have been implicated as being crucial in the progression of DM and its associated complications,^54^, ^55^, ^56^, ^57^, ^58^ which mainly lead to pancreatic β‐cell injury and IR (Figure 1). However, research on exosomal miRNAs is scant. We searched PubMed, Web of Science, and Embase using the following search terms: “Diabetes Mellitus” or “Diabetic” or “Diabetes” and “Exosome” or “Exosomal” and “miRNA” or “microRNA.” Meta‐analyses, reviews, case reports, comments, letters, and duplicate publications were excluded. A total of 106 papers published up to March 15, 2020 were included. They addressed the relationships of exosomal miRNAs with DM. The number of relevant studies increased markedly from 2015, reflecting the increasing interest in the relationships between exosomal miRNAs and DM.

## 4 MECHANISM OF EXOSOMAL miRNAS IN DM PROGRESSION

### 4.1 Exosomal miRNAs and pancreatic β‐cell injury

Pancreatic islets are composed of hormone‐releasing cells. Approximately 70% of pancreatic cells are β‐cells. Pancreatic β‐cells play a central role in maintaining blood glucose homeostasis through insulin release.^59^ β‐cell injury or dysfunction leads to the progression of DM.^60^ β‐cell injury typically occurs in the early pre‐diabetes stage. The three main mechanisms underlying β‐cell injury are hyperglycemia‐islet β‐cell injury, free fatty acid β‐cell damage, and amylin and β‐cell apoptosis.^59^ Recent studies have demonstrated that the enrichment of certain exosomal miRNAs can regulate related genes that have an important maintenance effect on pancreatic β‐cell homeostasis in the early stages of DM. In contrast, long‐term exposure to high concentrations of glucose and fatty acids negatively regulates the expression of these exosomal miRNAs. Therefore, enrichment of exosome‐specific miRNAs is involved in β‐cell dysfunction or injury in DM.^61^, ^62^, ^63^ Fu et al.^64^ constructed an ICR mouse model with a mixture of cytokines, including tumor necrosis factor‐beta (TNF‐β), interleukin‐1 (IL‐1), and interferon‐beta (INF‐β) and used streptozotocin (STZ) in vitro to induce injury. The authors isolated pancreatic islet tissue and performed exosomal miRNA sequencing. The results revealed a significant change in exosomal miR‐375‐3p in the two induced injury models and implicated miR‐375‐3p as a marker of islet injury. Exosomal miRNAs derived from other cells can also act on β‐cells. For example, exosomes containing miRNAs secreted by Min6B1 pancreatic cells treated with cytokines (IFNγ, TNF‐α, and IL‐1β) can be transferred to adjacent β‐cells, leading to the apoptosis of these cells.^54^ Tsukita et al.^65^ screened for miRNAs, the levels of which were significantly changed in the serum exosomes of mice after bone marrow transplantation (BMT) using a high‐throughput technique. Forty‐two miRNAs were upregulated. Of these, miR‐106b‐5p and miR‐222‐3p were secreted by bone marrow cells and transferred to pancreatic islet cells and induced β‐cell regeneration. Corresponding miRNA antagonist treatment inhibited BMT‐induced β‐cell regeneration. In addition, a tail vein injection of agomir miR‐106b‐5p and miR‐222‐3p into mice promoted the proliferation of injured β‐cells by downregulating the Cip/Kip family and thus improving hyperglycemia in insulin‐deficient DM mice. Interestingly, recent studies have found that exosomal miRNAs can be secreted by β‐cells and transferred to other receptor cells in addition to regulating β‐cell activity. Xu et al.^66^ confirmed in mouse models that exosomal miR‐26a derived from β‐cells can be transferred to mouse liver, white fat, brown fat, and other target organs and can be absorbed by the receptor hepatocyte. This transfer of exosomal miR‐26a results in improved insulin sensitivity of the receptor cells and maintenance of metabolic homeostasis. Furthermore, Xu et al.^67^ found that serum circulating miR‐204 was closely associated with the pancreatic β‐cell injury, which could be served as a novel biomarker for the early T1DM. The findings indicate that exosomal miRNAs are closely related to β‐cell damage and dysfunction in DM.

### 4.2 Exosomal miRNAs and IR

IR is the weakened response of cells to insulin. In this condition, normal levels of insulin cannot maintain normal glucose homeostasis. IR is a pathological state in which target tissues or cells have decreased sensitivity or reactivity to insulin. It is a hallmark of T2DM. IR mainly occurs in cells, such as fat cells, muscle cells, and liver cells, which rely on insulin to absorb glucose.^68^ The process of IR is associated with defects in insulin signaling, which involves the insulin receptor,^69^ insulin receptor substrate 1/2 (IRS‐1/2),^70^ glucose transporter 4 (GLUT4),^71^ and phosphoinositide 3‐kinase (PI3K)/AKT serine/threonine kinase (AKT).^72^ The mechanism underlying IR involves inflammation, oxidative stress, and autophagy. Recent studies have shown that miRNAs in exosomes participate in the mechanism underlying IR.^73^, ^74^, ^75^, ^76^, ^77^, ^78^ Katayama et al.^79^ found that miR‐20b‐5p is highly expressed in exosomes of T2DM patients and regulates glucose metabolism in human skeletal muscle cells through AKT signaling, thereby regulating the occurrence of IR. Consistent with these findings, another study showed that pancreatic cancer‐derived exosomal miR‐151‐3p and miR‐450b‐3p enter mouse myoblast C2C12 cells and inhibit PI3K/AKT signaling, thereby maintaining insulin‐induced FoxO1 rejection and inhibiting Glut4 transport.^80^ In addition, studies have shown that exosomal miR‐320a and miR‐27a are related to metabolic syndrome and T2DM. Exosomal miR‐509‐5p, miR‐23a, and miR‐197 are potential causes of dyslipidemia in metabolic syndrome.^81^ Exosomal miRNAs are closely related to aging‐related IR. Su et al.^55^ found that exosomal miR‐29b‐3p derived from bone marrow mesenchymal stem cells (BM‐MSCs) can regulate senescence‐induced IR, and may be a potential therapeutic target for aging‐related IR. Obesity is another important high‐risk factor for IR. In one study, obesity altered the expression profile of plasma exosomal miRNAs in mice. The authors reported that compared with that in lean mice, the expression of plasma exosomal miRNAs, including miR‐27b‐3p miR‐122, miR‐27a‐3p, and miR‐192, in obese mice was increased, and that glucose tolerance and IR were induced in lean mice treated with exosomes isolated from obese mice.^82^ Ying et al.^83^ obtained exosomes of adipose tissue macrophages (ATM) from obese mice and transferred them to lean mice. This also resulted in glucose intolerance and IR in the lean mice. Conversely, ATM exosomes obtained from lean mice and transferred to obese mice improved glucose tolerance and insulin sensitivity in the obese mice. The mechanism is related to ATM exosomal miR‐155, which regulates the occurrence of IR by targeting the expression of the peroxisome proliferator‐activated receptor gamma gene. Collectively, these findings substantiate the important role of exosomal miRNAs in the pathological process of IR.

## 5 ROLES OF EXOSOMAL MIRNAS IN DIABETIC COMPLICATIONS

**FIGURE 2:**
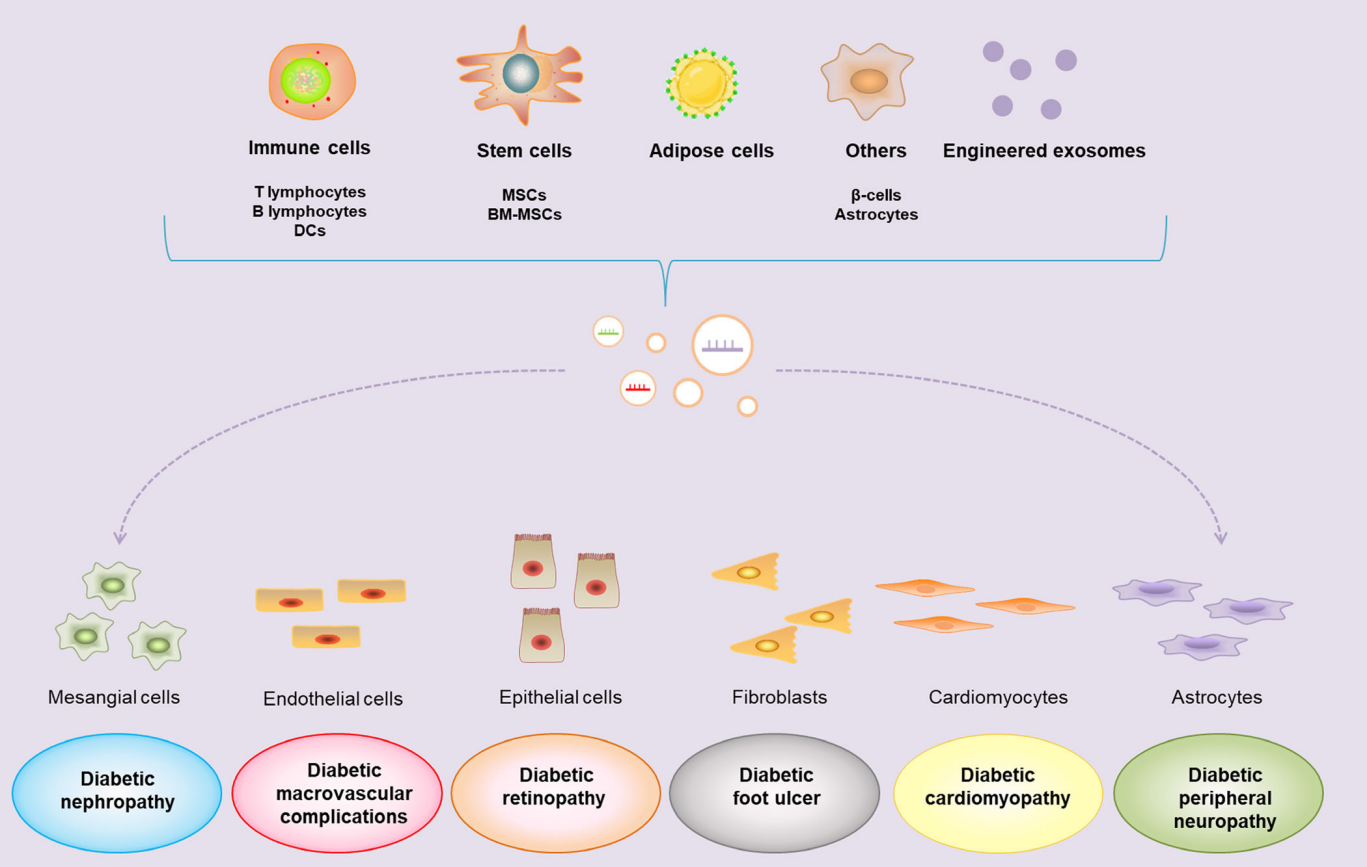
Relationship between exosomal miRNAs and cell events of DM‐associated complications. BM‐MSCs, bone marrow mesenchymal stem cells; DCs, dendritic cells; MSCs, mesenchymal stem cells

**FIGURE 3:**
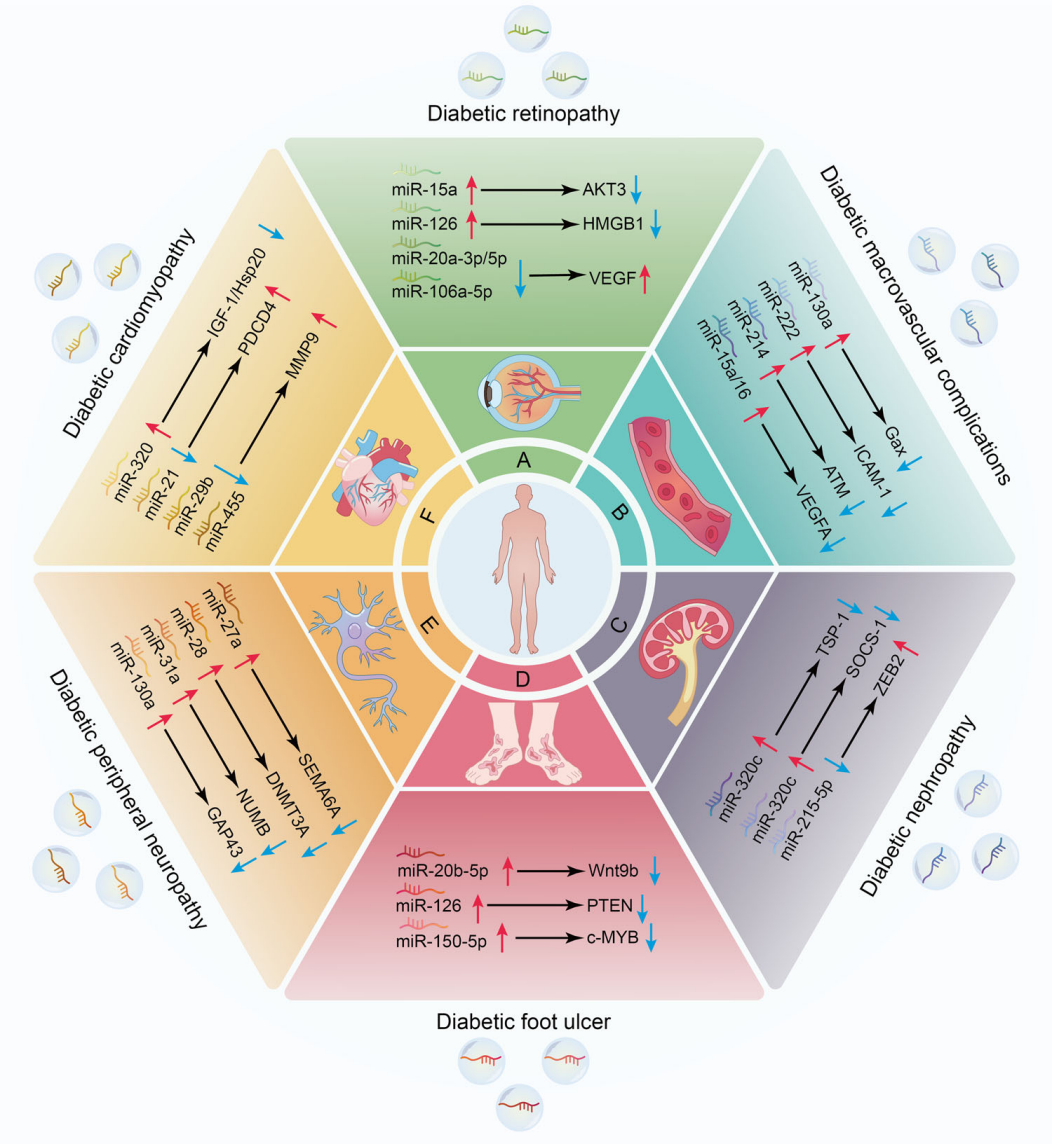
Molecular mechanisms by which exosomal miRNAs regulate the progression of DM‐associated complications. In the diabetic condition, exosomal miRNAs are taken up by recipient cells, where they exert their biological function and thereby modulate the progression of DM‐associated complications, including diabetic retinopathy (DR) (A), diabetic macrovascular complications (DMCs) (B), diabetic nephropathy (DN) (C), diabetic foot ulcer (DFU) (D), diabetic peripheral neuropathy (DPN) (E), and diabetic cardiomyopathy (DCM) (F). AKT3, AKT serine/threonine kinase 3; ATM, ataxia telangiectasia mutated; c‐MYB, MYB proto‐oncogene; DNMT3A, DNA methyltransferase‐3α; GAP43, growth‐associated protein‐43; Gax, mesenchyme homeobox 2; HMGB1, high‐mobility group box 1; Hsp20, heat shock protein 20; ICAM‐1, intercellular adhesion molecule‐1; IGF‐1, insulin like growth factor 1; MMP9, matrix metalloproteinase 9; NUMB, NUMB endocytic adaptor protein; PDCD4, programmed cell death 4; PTEN, phosphatase and tensin homolog; SEMA6A, semaphorin 6A; SOCS‐1, suppressor of cytokine signaling 1; TSP‐1, thrombospondin 1; VEGF, vascular endothelial growth factor; VEGFA, vascular endothelial growth factor‐A; Wnt9b, Wnt family member 9B; ZEB2, zinc finger E‐box binding homeobox 2

Recent studies showed that exosomal miRNAs are closely associated with the progression of DM and its associated complications, which are involving in a series of cell events (Figure 2), thereby forming a complex regulatory network (Figure 3).

### 5.1 Exosomal miRNAs and DN

DN is a major complication of DM and is the leading cause of end‐stage renal disease in DM. DN is one of the leading causes of disability and death in diabetic patients.^84^, ^85^ It is characterized by progressive renal interstitial fibrosis that involves a series of pathological changes, including excessive accumulation of extracellular matrix, mesangial expansion, thickening of glomerular and tubular basement membranes, and increased production of mesangial matrix.^86^

Recent studies have reported that the levels of DN‐derived exosomal miRNAs are associated with the clinical characteristics of DN. Exosomal miRNAs in urine have been widely used to analyze the correlated clinical characteristics of DN patients. This is because urine can be conveniently collected and used to assess specific constituent molecules of interest. Diabetic proteinuria is an important marker for the diagnosis of DN and can reflect the pathogenesis of DN.^87^ Recent studies have closely related exosomal miRNAs in the urine of DN patients with urinary protein content. Eissa et al.^88^ found that the expression of miR‐30a, miR‐342, and miR‐133b in urine exosomes from T2DN patients was significantly higher than that in healthy people and that the high expression of these three miRNAs was associated with HbA1c, systolic, diastolic, low‐density lipoprotein, serum creatinine, estimated glomerular filtration rate (eGFR), and urinary albumin creatinine ratio. In addition, these three types of urine exosomal miRNAs in T2DN with normal urinary albumin were significantly changed before the appearance of proteinuria. Interestingly, Jia et al.^89^ demonstrated that the urine exosomal miR‐192 levels in T2DM patients with microalbuminuria were higher than those in T2DM patients with normoalbuminuric and healthy controls, and that urine exosomal miR‐192 levels were positively correlated with albuminuria levels and transforming growth factor (TGF)‐β1 expression. The findings inform a strategy in which urinary exosomal miR‐192 is used as a biomarker of early‐stage DN. In addition, urinary exosomal miRNAs can also be used as biomarkers to monitor the condition and therapeutic effect of T2DM‐related nephropathy. Delic et al.^90^ reported that both telmisartan and lnagliptin restored the levels of urinary exosomal miR‐29c to normal. This miRNA plays an antifibrotic role in nephrectomized rat models.

Exosomal miRNAs have potential as biomarkers for the diagnosis and monitoring of therapeutic effects in DN patients. Animal and cell models of DN also confirmed the important roles of exosomal miRNAs in the pathogenesis of nephropathy. Jin et al.^91^ demonstrated that adipose‐derived stem cell‐derived exosomal miR‐215‐5p effectively inhibited the migration and apoptosis of podocytes induced by elevated glucose by directly targeting the expression of ZEB2, thereby improving DN induced by podocyte injury. Lv et al.^92^ described that tubular epithelial cell‐derived exosomal miR‐19b‐3p was internalized by macrophages, leading to M1 phenotype polarization by directly targeting the expression of suppressor of cytokine signaling 1 (SOCS‐1), causing nuclear factor‐kappa B (NF‐κB) signaling‐associated tubulointerstitial inflammation to induce DN progression. Delić et al.^93^ used high‐throughput sequencing to analyze the differential expression of exosomal miRNAs between the urines of T2DM patients with DN and healthy individuals. The authors found that the significantly increased exosomal miR‐320c in the urine of DN patients may target thrombospondin 1 to affect the TGF‐β signaling pathway and thus play a regulatory role in the DN process. These results indicate that exosomal miRNAs are important for the diagnosis and treatment of T2DM‐related kidney diseases. They are expected to become new candidate markers and therapeutic targets for DN.

### 5.2 Exosomal miRNAs and DMCs

DMCs usually involve organs such as the heart and brain, and are accompanied by pathological changes in endothelial cells (ECs), cardiomyocytes, vascular cells, and stem cells. DMCs are important factors in DM‐related death. DMCs usually occur early in the early stage of DM, and high levels of glucose in the blood can lead to endothelial dysfunction and microvascular sparseness.^94^, ^95^ The occurrence and development of DMCs involves various mechanisms, including autophagy,^96^ inflammatory response,^97^, ^98^ oxidative stress,^99^, ^100^ and immune response.^101^ These mechanisms lead to vascular calcification and vascular endothelial injury, which ultimately lead to DMCs.

On the contrary, exosomal miRNAs play an important role in the process of DMC‐related vascular endothelial disease. Mocharla et al.^102^ revealed that CD34(+) peripheral blood mononuclear cell (PBMC)‐derived exosomal miR‐126 could increase the proangiogenic effects of PBMCs, thereby inhibiting the impaired proangiogenic effects due to elevated levels of glucose in DM. DM is often accompanied by atherosclerosis, and DMC‐related vascular endothelial lesions are closely related to atherosclerosis. van Balkom et al.^103^ demonstrated that EC‐derived exosomal miR‐214 suppresses senescence and stimulates blood vessel formation in neighboring target ECs by directly repressing the expression of the target gene (ataxia telangiectasia mutated), which may be related to DMC endothelial lesions. Interestingly, Hergenreider et al. found that exosomal miR‐143/145 induced by the shear‐responsive transcription factor Krüppel‐like factor 2 (KLF2) could mediate communication between ECs and vascular smooth muscle cells (VSMCs). The authors also showed that miR‐143/145 directly inhibited the expression of target genes (e.g., *CFL1*, *PHACTR4*, and *SSH2*) in VSMCs, thereby preventing the pathogenesis of vascular ECs.^104^ Moreover, the increase in circulating endothelial microparticles (EMPs) was closely associated with DM‐associated atherosclerosis.^105^ Jansen et al.^106^ reported that EMPs could transfer miR‐222 into recipient ECs and promote anti‐inflammatory effects in vitro and in vivo by inhibiting the expression of its target gene, intercellular adhesion molecule (ICAM)‐1. However, EMPs derived from high glucose‐damaged ECs contain less miR‐222 and have weaker anti‐inflammatory capacity, which may provide a novel therapeutic strategy for DMC‐related coronary artery disease.

On the contrary, exosomal miRNAs are also associated with DMC‐related angiogenesis deficiencies, while insufficient neovascularization is the main factor in ischemic diseases caused by DM. Spinetti et al.^107^ demonstrated that exosomal miR‐15a and miR‐16 were absorbed in circulating proangiogenic cells, resulting in an impaired migratory capacity of PACs by directly inhibiting the expression of AKT‐3 and vascular endothelial growth factor‐A (VEGFA), thereby increasing the risk of critical limb ischemia in T2DM patients.^107^ Loss of skeletal muscle capillarization (also known as capillary rarefaction) is a common symptom of T2DM. Nie et al.^108^ found that skeletal muscle‐derived exosomes contain angiogenic miR‐130a and that their transfer to human umbilical vein endothelial cells (HUVECs) enhances the production of reactive oxygen species and angiogenesis of HUVECs by inhibiting the expression of target gene mesenchyme homeobox 2 (Gax). These events relieve the T2DM‐induced loss of skeletal muscle capillarization. Collectively, these findings indicate that exosomal miRNAs can provide effective treatment for DMC‐related endothelial injury and insufficient neovascularization.

### 5.3 Exosomal miRNAs and DR

DR is a common complication of DM and is the leading cause of catastrophic vision loss in industrialized nations.^109^, ^110^ Persistent hyperglycemia can damage retinal microvessels, leading to increased permeability, reduced retinal blood flow, and even microvessel closure, which ultimately leads to retinopathy.^111^, ^112^ DR‐induced visual deterioration is usually accompanied by neovascularization, vascular hyperpermeability, inflammation, and vascular cell dysfunction.^113^, ^114^ DR has obvious pathological and clinical characteristics. Nonetheless, it is important to search for more effective biomarkers for the monitoring and therapy of DR, because DR has a complex pathogenesis and ambiguous risk factors.^115^, ^116^

Recent studies reported the important role of exosomal miRNAs in the development of DR, especially in retinal cell dysfunction. For example, Kamalden et al.^117^ demonstrated that the pancreatic β‐cell‐derived exosomal miR‐15a can enter the bloodstream and promote the apoptosis of retinal Müller cells by targeting Akt3, which may lead to retinal injury. In addition, Maisto et al.^118^ reported that elevated levels of glucose decreased the levels of antiangiogenic miRNAs (e.g., miR‐106a‐5p, miR‐20a‐5p, and miR‐20a‐3p) in exosomes released by primary retinal cells to regulate the expression of VEGF, thereby promoting damage to retinal photoreceptors. Interestingly, other studies have shown that exosomal miRNAs secreted by MSCs can effectively alleviate the relevant characterization of DR, which may provide a novel strategy for the prevention and treatment of DR. Zhang et al.^119^ reported that miR‐126 could be encapsulated in MSC‐derived exosomes and transferred to human retinal ECs (HRECs). MSC‐exosome‐derived miR‐126 significantly decreased the HMGB1 expression induced by elevated glucose and restricted the inflammatory response in HRECs. Moreover, Safwat et al.^120^ revealed that MSC‐derived exosomal miR‐222 was closely associated with regenerative changes in the retina in a rabbit model of DR, which suggests that exosomal miR‐222 plays a vital role in the process of retinal tissue repair. In future DR treatment, exogenously increasing the content of certain exosomal miRNAs in the human body could improve the state of retina‐related cells.

### 5.4 Exosomal miRNAs and DFU

DM can affect blood vessels throughout the body. When it involves the skin, it can manifest as poor wound healing in the form of DM‐related ulcers, which mainly occur in the feet of diabetic patients. DFU is a severe complication of DM, which affects 15% of diabetic patients and leads to a risk of amputation, and even high mortality rates.^121^ Epidemiological findings indicate that the 5‐year mortality rates of patients with DFU are 2.5 times higher than those of patients without DFU.^122^ Peripheral arterial disease is one of the major causes of DFU, leading to foot ulcerations and resulting in nonhealing ulcerations.^123^

Recently, increasing evidence suggests that exosomal miRNAs function critically in regulating the progress of DFU. Xiong et al.^124^ used high‐throughput sequencing analysis to demonstrate the upregulation of circulating exosomal miR‐20b‐5p in the peripheral blood of T2DM patients compared to that of healthy controls. The authors also revealed that exosomal miR‐20b‐5p derived from T2DM suppressed wound healing in a mouse model in vivo by inhibiting the Wnt9b/β‐catenin signaling pathway. In addition, the occurrence and development of DFU is often accompanied by an inflammatory response and abnormal expression of inflammatory factors. Geiger et al.^125^ revealed that human circulating fibrocyte‐derived exosomes contain large amounts of angiogenic miRNAs (miR‐132, miR‐130a, and miR‐126), anti‐inflammatory miRNAs (miR‐125b and miR124a), and miR‐21, which regulate collagen deposition. These miRNAs can accelerate wound closure by activating diabetic dermal fibroblasts and promoting the migration and proliferation of diabetic keratinocytes in diabetic mice, thereby accelerating DM‐associated wound healing. Exosomal miRNAs are also important in the treatment of DFU. For example, exosomes secreted by MSCs can promote the healing of diabetic wounds caused by autotransplantation.^126^, ^127^ Ding et al.^128^ found that exosomes from bone marrow‐derived MSCs preconditioned by deferoxamine could stimulate angiogenesis in HUVECs and promote wound healing and angiogenesis in STZ‐induced diabetic rats by affecting exosomal miR‐126/PTEN/PI3K/AKT signaling. Henriques‐Antunes et al.^129^ used a light‐triggerable hydrogel containing exosomes to treat diabetic and nondiabetic wounds with one dose, and demonstrated that the kinetics of exosome delivery affect skin neovascularization and re‐epithelization in a mouse model of T1DM by altering the expression of exosomal miR‐150‐5p. These results suggest that exosomal miRNA has the potential to treat diabetic ulcers. By artificially encapsulating miRNA agonists or inhibitors into exosomes or other nanomaterials, followed by local injection into the diabetic ulcer tissue or intravenous injection, exosomes can be used as a potential drug delivery system to treat diabetes‐related DFU.

### 5.5 Exosomal miRNAs and DCM

DCM is a condition in which myocardial function is impaired due to DM. DCM is defined as myocardial dysfunction occurring in patients with DM in the absence of coronary artery disease, hypertension, or valvular heart disease.^130^, ^131^ DCM usually carries a substantial risk for subsequent heart failure and increased mortality.^132^ The pathogenesis of DCM may involve oxidative stress, inflammation, impaired calcium handling, cardiomyocyte apoptosis, mitochondrial dysfunction, and renin‐angiotensin system activation.^133^

Recently, increasing evidence has demonstrated that exosomal miRNAs in cardiomyocytes are closely related to DCM‐related myocardial injury and myocardial angiogenesis disorders.^134^, ^135^, ^136^ Wang et al.^137^ separated cardiomyocytes from adult Goto–Kakizaki (GK) rats, a commonly used animal model of T2DM, and found that GK cardiomyocyte‐derived exosomal miR‐320 inhibits the proliferation and migration of mouse cardiac ECs (MCECs) by targeting the expression of Ets2, Hsp20, and IGF‐1, thereby causing DM‐induced myocardial vascular deficiency. Garcia et al.^138^ demonstrated that glucose starvation increases the secretion of cardiomyocyte‐derived exosomes containing miRNAs (e.g., miR‐126‐3p and miR‐23a), thereby promoting the angiogenesis of HUVECs. The communication between cardiomyocytes and HUVECs may affect DCM‐associated cardiac injury and repair. Chaturvedi et al.^139^ demonstrated that exercise could increase the levels of cardiomyocyte‐derived exosomal miR‐455 and miR‐29b in a db/db mouse model and reduce the expression of the miR‐455 and miR‐29b target gene matrix metalloproteinase 9 (MMP9). This gene plays a role in matrix degradation and leads to fibrosis and myocyte uncoupling, thereby relieving diabetic heart complications. Exosomal miRNAs secreted by stem cells and other types of cells (including cardiomyocytes) play an important role in heart cell regeneration and cardiac function regulation.^140^, ^141^, ^142^ Xiao et al.^143^ found that cardiac progenitor cell‐derived exosomal miR‐21 could suppress H9C2 cardiac cell apoptosis induced by oxidative stress by inhibiting the expression of the target gene, programed cell death 4 (PDCD4). This inhibition protected the myocardial cells against oxidative stress‐related apoptosis. These findings indicate that exosomes containing miR‐21 may be used as a new therapeutic vehicle for DCM‐associated ischemic cardiac disease. The use of exosome secretion inhibitors (e.g., GW4869) may also be a potential therapeutic strategy to alleviate exosome‐mediated diabetic cardiac dysfunction.^144^, ^145^ These findings collectively indicate that exosomal miRNAs may be potentially valuable in the treatment of DCM.

### 5.6 Exosomal miRNAs and diabetic peripheral neuropathy (DPN)

DPN is one of the most common chronic complications of T2DM. It is estimated that nearly 50 million people worldwide will develop DPN by 2030.^146^, ^147^ DPN has typical symptoms of nerve pathological pain, including spontaneous pain, allodynia (pain to normally innocuous stimuli), and hyperalgesia (increased pain perception to noxious stimuli).^148^ Clinical studies of T2DM have revealed that glucose control has little or no effect in alleviating DPN and that DPN has become a substantial problem in the intractable pain therapy of DM.^149^, ^150^, ^151^ Effective therapies to improve neurological function and alleviate DPN‐associated damage to the peripheral nervous system are urgently needed.

Several recent studies have indicated that exosomal miRNAs play an important role in a diabetic DPN rat model, which is closely related to the occurrence and development of DPN. Jia et al.^152^ reported exosomal miRNAs (e.g., miR‐28, miR‐31a, and miR‐130a) derived from Schwann cells stimulated by elevated glucose could communicate with axons of dorsal root ganglia (DRG). These exosomal miRNAs were locally injected into the sciatic nerves of diabetic db/db mice, where they could promote the occurrence and development of peripheral neuropathy by regulating the expression of DNA methyltransferase‐3α (DNMT3A), synaptosome‐associated protein 25 (SNAP25), NUMB, and growth‐associated protein‐43 (GAP43). Interestingly, Wang et al.^153^ demonstrated that healthy Schwann cell‐derived exosomal miRNAs (e.g., miR‐21, miR‐27a, and miR‐146a) could ameliorate DPN in type II diabetic db/db mice by promoting the neurite outgrowth of diabetic DRG neurons. In addition, exosomes secreted by MSCs can repair damaged neurons and astrocytes and reverse neurological dysfunction. Fan et al.^56^ reported that exosomes derived from mesenchymal stromal cells (e.g., miR‐17, miR‐23a, miR‐125b) markedly decrease the threshold for thermal and mechanical stimuli and increase the nerve conduction velocity in diabetic mice. These events specifically alleviated neurovascular dysfunction in mice with DPN by inhibiting the toll‐like receptor (TLR) 4/NF‐κB signaling pathway and suppressing the expression of proinflammatory proteins. These results suggest that exosomal miRNAs are an effective treatment tool for diabetic nerve injury.

## 6 POTENTIAL CLINICAL APPLICATIONS OF EXOSOMAL MIRNAS IN DM

Because DM is a class of chronic metabolic disorder diseases, the therapeutic effect of exosomal miRNAs in DM patients can be improved via its early detection by timely intervention, with particular attention to glycemic control, blood pressure control (thus limiting proteinuria), and accentuation of cardiovascular risk.^7^ Therefore, there is great urgency for identifying novel molecular markers and drug targets of DM. Recently, an increasing number of studies have shown that the expression of exosomal miRNAs changes during the progression of DM and its associated complications. Because of their unique structure, exosomal miRNAs are more stable in tissues and cells and are development phase‐specific.^154^ Exosomal miRNAs are easier to extract and can be detected with higher specificity compared with proteins. Detecting exosomal miRNAs using qRT‐PCR and in situ hybridization assays is also more specific and sensitive than detecting proteins using an antigen–antibody reaction.^155^ Recently, substantial evidence has indicated that the expression profiles of many exosomal miRNAs vary between the sera and urines of healthy individuals and those with DM. Therefore, exosomal miRNAs may serve as novel diagnostic biomarkers of DM (Table 1). Using miR‐1 and miR‐133a as diagnostic biomarkers,^158^ receiver operating characteristic (ROC) curve analysis showed that miR‐1 and miR‐133a expression levels are good candidates to distinguish between diabetic and nondiabetic serum samples (sensitivity 78.9% and 78.9%, specificity 71.0% and 74.2%, respectively). The area under the ROC curve (AUC) values were 0.886 (95% confidence interval [CI]: 0.765–0.967, *p *< .001) and 0.825 (95% CI: 0.710–0.940, *p *< .001), respectively. Moreover, urinary exosomal miRNAs displayed similar diagnostic values. Eissa et al.^88^ described an ROC curve analysis indicating that implicated miR‐30a, miR‐342, and miR‐133b expression levels were good candidates to distinguish between diabetic and nondiabetic serum samples (sensitivity 86.4%, 81.8%, and 76.4%, specificity 72.7%, 80.9%, and 90.9%, respectively). The AUC values were 0.867 (95% CI: 0.820–0.914, *p *< .001), 0.910 (95% CI: 0.873–0.948, *p *< .001), and 0.897 (95% CI: 0.858–0.936, *p *< .001), respectively. Furthermore, Sidorkiewicz et al.^162^ demonstrated that exosomal miR‐491‐5p, miR‐1307‐3p, and miR‐298 can be used as novel biomarkers for predicting the progression from prediabetes to T2DM, and the AUC values were 0.940, 0.880, and 0.840, respectively. Interestingly, exosomal miRNAs are closely associated with the gender difference of DM. Deng et al.^163^ found that serum exosomal miR‐29a and miR‐29b displayed the diagnostic values for the pregnant women with GDM, and ROC curve analysis indicated that miR‐29a combined with miR‐29b (the AUC value was 0.944 [95% CI: 0.907–0.982]) for the diagnosis was prior than the single indicator (the AUC values of miR‐29a and miR‐29b were 0.829 [95% CI: 0.755–0.903] and 0.857 [95% CI: 0.787–0.926]). Thus, exosomal miRNAs are promising molecular biomarkers for the screening and monitoring of DM.

**TABLE 1:** Diagnostic index of exosomal miRNAs in DM

Exosomal miRNAs	Types	Sample numbers (nondiabetic/diabetic)	AUC	Sensitivity (%)	Specificity (%)	OR (95% CI)	Ref.
miR‐133b	T2DN	56/110	0.867	86.4	72.7	0.820–0.914	^88^
miR‐342	T2DN	56/110	0.910	81.8	80.9	0.873–0.948	^88^
miR‐30a	T2DN	56/110	0.897	76.4	90.9	0.858–0.936	^88^
miR‐15b	T2DM	44/136	0.883	97.8	82.2	0.824–0.942	^156^
miR‐34a	T2DM	44/136	0.917	93.3	86.7	0.874–0.96	^156^
miR‐636	T2DM	44/136	0.984	97.8	93.3	0.971–0.997	^156^
miR‐7	T2DM	74/76	0.75	—	—	0.670–0.830	^157^
miR‐7	T2DMC	74/76	0.77	—	—	0.690–0.85	^157^
miR‐133a	T2DM	12/78	0.825	78.9	74.2	0.710–0.940	^158^
miR‐1	T2DM	12/78	0.886	78.9	71.0	0.765–0.967	^158^
miR‐21‐5p	T2DN	15/22	0.830	–	–	0.673–0.986	^159^
miR‐30b‐5p	T2DN	15/22	0.714	–	–	0.517–0.911	^159^
let‐7c‐5p	T2DN	15/28	0.818	96	53.4	0.718–0.919	^160^
miR‐424	T1DM	30/30	0.803	–	–	–	^161^
miR‐218	T1DM	30/30	0.817	–	–	–	^161^
miR‐491‐5p	Prediabetes	42/24	0.940	–	–	–	^162^
miR‐1307‐3p	Prediabetes	42/24	0.880	–	–	–	^162^
miR‐298	Prediabetes	42/24	0.840	–	–	–	^162^
miR‐29a	GDM	55/68	0.829	76.47	78.18	0.755–0.903	^163^
miR‐29b	GDM	55/68	0.857	85.29	81.82	0.787–0.926	^163^

*Note*: GDM, gestational diabetes mellitus; T1DM, type 1 diabetes mellitus; T2DM, type 2 diabetes mellitus; T2DMC, T2DM‐associated microvascular complications; T2DN, type 2 diabetic nephropathy.

Exosomal miRNAs cannot only be used as biomarkers to gauge the progress of DM, but can also be used as therapeutic targets for DM. Exosomes can be served as a new type of nanomaterial and can deliver miRNA inhibitors and agonists for DM treatment. The widespread application of nanotechnology has prompted the use of exosomal miRNAs in animal experiments. Lv et al.^164^ used electroporation to mimic the loading of miR‐21‐5p mimics into human adipose stem cell (hASC)‐derived exosomes. The authors showed that engineered exosomal miR‐21‐5p could increase re‐epithelization, vessel maturation, angiogenesis, and collagen remodeling to accelerate wound healing in diabetic rats with full‐thickness wounds. These findings indicate the possibility of cell‐free therapy for diabetic wounds using hASC exosomes to deliver drugs. Li et al.^165^ used synovial MSC (SMSC)‐derived exosomal miR‐126‐3p encapsulated in hydroxyapatite/chitosan composite hydrogels as wound dressings for a mouse model of diabetic wounds. The authors reported that the released SMSC exosomal miR‐126‐3p nanoparticles could promote wound surface re‐epithelialization, accelerate angiogenesis, and expedite collagen maturity in vivo. These findings could inform the development of a novel therapeutic strategy for diabetic chronic wound healing. Shi et al.^166^ used diabetic pregnant mouse models to demonstrate that fluorescently labeled exosomes in blood can carry miRNAs across the placental barrier and may penetrate embryonic organs and tissues, including the heart, during embryonic development. This could increase the risk of coronary heart disease in normal recipient pregnant mice. These findings may inform a new strategy for the prevention and treatment of coronary heart disease. Studies of corresponding specific exosome inhibitors or agonists indicate a potential therapeutic strategy for alleviating exosome‐mediated diabetes and related complications. However, the path from basic scientific research to clinical application remains lengthy, and the use of exosomal miRNAs for DM treatment remains challenging. Further research in the development of biomedical materials technology will clarify the function and mechanism of exosomal miRNAs and should lead to novel strategies for the screening, early diagnosis, and therapy of DM.

## 7 OPPORTUNITIES AND CHALLENGES OF EXOSOMAL MIRNAS IN DM

With the development of next‐generation sequencing technologies,^167^ an increasing number of exosomal miRNAs have been discovered and identified. Compared with exosomal proteins, miRNA extraction and detection have higher specificity and sensitivity. Recently, a few databases have been developed as convenient tools for identifying and predicting exosomal components (proteins, miRNAs, mRNAs, and lipids) (Table 2). Among them, CMEP, Xeno‐miRNet, miRandola, and other databases can be used to mine disease‐related exosomal miRNAs. In the recent years, with the development of biological sciences and materials technology, the nanotechnology is widely used in the field of disease treatment,^178^, ^179^ including DM.^180^ Different from taking the agomir or antagomir directly injecting into target tissues, nanocarrier‐derived miRNAs displayed more high efficiency and target specificity,^181^ which may merit for being developed as a therapeutic strategy for DM. Exosomes as endogenous nanocarriers can be used in target therapy via delivering drug cargos to the disease‐associated targeted cells, which has advantages such as multiple drug loading, lack of toxicity, harboring a high payload of drugs, and protecting contents from drug degradation.^182^In view of the important role of exosomal miRNAs in the progression of DM and DM‐associated complications, the approach has great potential as a biological tool for the diagnosis or treatment of DM. However, much remains to be done before the research findings become clinical reality.

**TABLE 2:** Exosomal miRNA‐associated databases

Database	Functions of database	Website	Ref.
EVpedia	It is a community web portal for systematic analyses of prokaryotic and eukaryotic EV‐related research	http://evpedia.info	^168^
ExoCarta	It catalogs information on the exosomal isolation and purification procedures, samples used, investigator details, and exosomal molecular components such as proteins, mRNA, and miRNA	http://www.exocarta.org/	^169^
Vesiclepedia	A manual search tool library of extracellular vesicle molecular data. It can also browse and search by species, vesicle, molecule, and sample type	http://www.microvesicles.org	^170^
EVmiRNA	It provides the miRNA expression profiles and the sample information of EVs from different sources, the specifically expressed miRNAs in different EVs and miRNA annotations	http://bioinfo.life.hust.edu.cn/EVmiRNA	^171^
BoMiProt	It is a manually curated, comprehensive repository of published information of bovine milk proteins, and focus efforts to consolidate the existing information of different milk proteins	http://bomiprot.org	^172^
CMEP	It contains large‐scale circulating miRNA datasets from diverse platforms and provides miRNA expression profiling, pathway enrichment analysis with miRNA target genes, and feature‐selection methods	http://syslab5.nchu.edu.tw/CMEP	^173^
Xeno‐miRNet	Search and explore xeno‐miRNAs and their potential targets within different host species	http://xeno.mirnet.ca	^174^
exoRBase	Aims to collect and characterize all long RNA species in human blood exosomes	http://www.exoRBase.org	^175^
NONCODEv5	Constructing human lncRNA‐disease relationships and single nucleotide polymorphism‐lncRNA‐disease relationships; displaying human exosome lncRNA expression profiles; predicting the RNA secondary structures of NONCODE human transcripts	http://www.noncode.org/	^176^
miRandola	To infer the potential biological functions of circulating miRNAs and their connections with phenotypes.	http://atlas.dmi.unict.it/mirandola/index.html	^177^

Research on exosomal miRNAs faces a series of challenges and limitations. First, the technology for isolation and purification of exosomes is not yet mature. At present, EVs can be separated and purified using ultrahigh‐speed centrifugation, filtration, precipitation, and immunoenrichment.^183^, ^184^ These purification methods cannot easily distinguish exosomes from nonvesicular compartments, which may affect the subsequent experiment of exosomal miRNAs in vivo and in vitro. Second, due to the low abundance of the exosomal miRNAs content in patients’ serum,^185^ there is lack of high‐efficiency method to collect exosomes in clinical application, which restricts the potential clinically relevant application of exosomal miRNAs as diagnostic and therapeutic markers. Third, exosomal miRNAs need to be further investigated to determine whether they are specifically related to one or more diseases, and to explore the underlying molecular mechanisms of exosomal miRNAs in diseases. Fourth, the exact mechanism and specific role of exosomal miRNAs in the regulation of DM need to be further clarified. Interestingly, Chevillet et al.^186^ posed a challenge to the hypothesis that all the presentation is about the natural transfer of miRNAs through exosomes. Fifth, it is difficult to develop an exosome‐based drug delivery system and introduce it into the body to target specific cells to play a functional role. Last, although exosomal miRNAs play a role in the treatment of DM and its complications in many animal models, there remains a lack of clinical trials to confirm the accuracy and safety of these findings.

## 8 CONCLUSIONS

**FIGURE 4:**
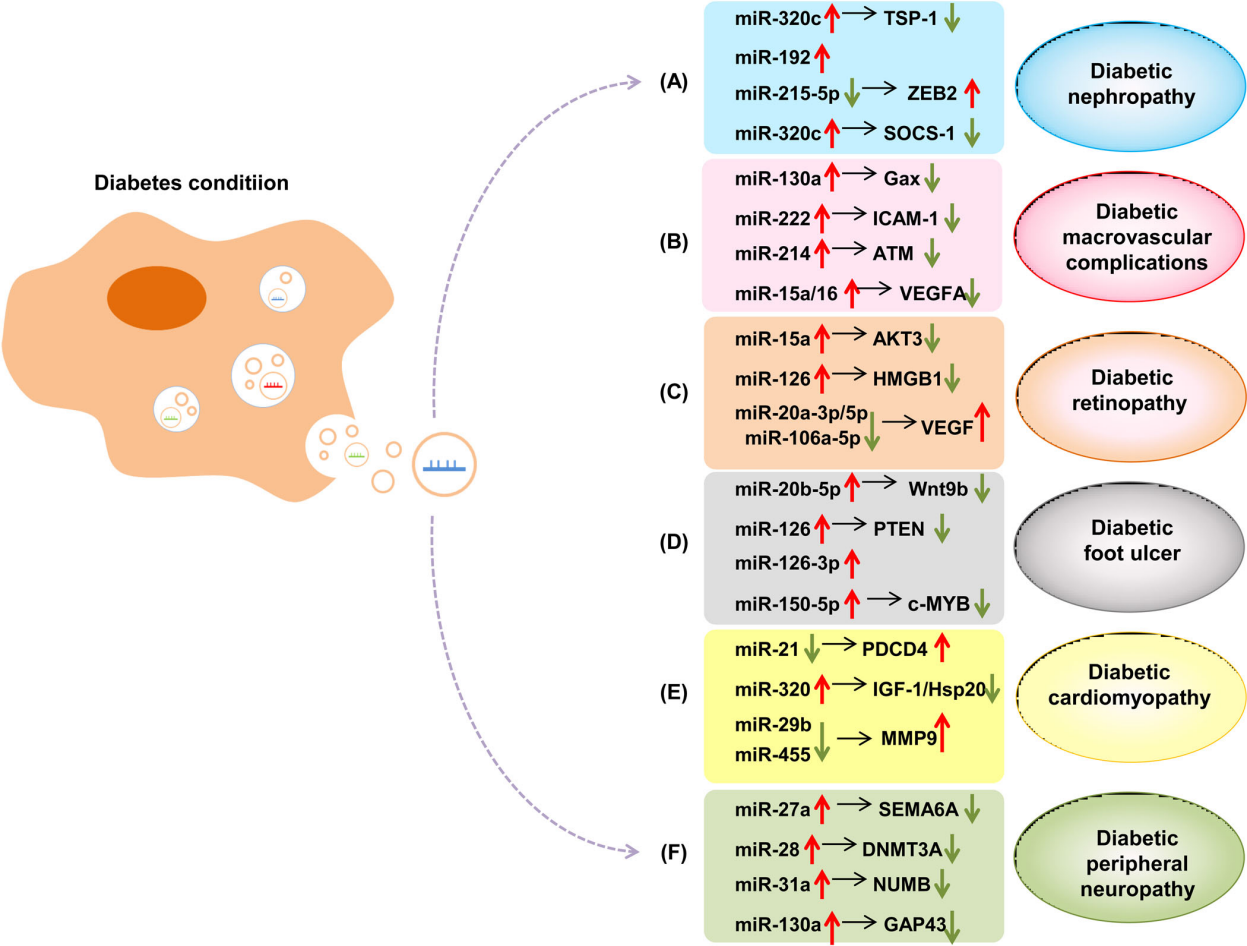
Relationship between exosomal miRNAs and DM‐associated complications. In the diabetic condition, exosomal miRNAs are taken up by recipient cells, where they exert their biological function and thereby modulate the progression of DM‐associated complications, including diabetic nephropathy (DN) (A), diabetic macrovascular complications (DMCs) (B), diabetic retinopathy (DR) (C), diabetic foot ulcer (DFU) (D), diabetic cardiomyopathy (DCM) (E), and diabetic peripheral neuropathy (DPN) (F)

Exosomal miRNAs are closely associated with the progression of DM and its associated complications (Figure 4). Therefore, exosomal miRNAs have been recognized as novel and potentially valuable molecules in DM research. As a class of novel regulatory molecules, they participate in multiple steps of DM by modulating the expression levels of a series of related genes. We can silence or activate exosomal miRNAs in DM patients exogenously, such as by incorporating agomir or antagomir into exosomes in vitro, followed by their injection into target tissues. This approach has merit for being developed as a therapeutic strategy for DM. Although our current understanding of the development of exosome‐based drug delivery systems is only the tip of the iceberg, with further research and technological advances, exosomal miRNAs are expected to lead to new strategies for the prevention, diagnosis, and treatment of DM.

## CONFLICT OF INTEREST

The authors declare that there is no conflict of interest.

## AUTHOR CONTRIBUTIONS

Xiaoyun He, Gaoyan Kuang, and Chunlin Ou designed/planned the study, performed imaging analysis, and wrote the paper. All authors participated in writing the paper.
